# Altered brain activities in mesocorticolimbic pathway in primary dysmenorrhea patients of long-term menstrual pain

**DOI:** 10.3389/fnins.2023.1098573

**Published:** 2023-01-30

**Authors:** Ni Liu, Yingqiu Li, Yueying Hong, Jianwei Huo, Tai Chang, Haoyuan Wang, Yiran Huang, Wenxun Li, Yanan Zhang

**Affiliations:** ^1^Department of Radiology, Beijing Hospital of Traditional Chinese Medicine, Capital Medical University, Beijing, China; ^2^School of Acupuncture-Moxibustion and Tuina, Beijing University of Chinese Medicine, Beijing, China

**Keywords:** primary dysmenorrhea, resting-state functional magnetic resonance imaging, regional homogeneity, amplitude of low-frequency fluctuations, functional connectivity

## Abstract

**Background:**

Patients with primary dysmenorrhea (PDM) often present with abnormalities other than dysmenorrhea including co-occurrence with other chronic pain conditions and central sensitization. Changes in brain activity in PDM have been demonstrated; however, the results are not consistent. Herein, this study probed into altered intraregional and interregional brain activity in patients with PDM and expounded more findings.

**Methods:**

A total of 33 patients with PDM and 36 healthy controls (HCs) were recruited and underwent a resting-state functional magnetic resonance imaging scan. Regional homogeneity (ReHo) and mean amplitude of low-frequency fluctuation (mALFF) analysis were applied to compare the difference in intraregional brain activity between the two groups, and the regions with ReHo and mALFF group differences were used as seeds for functional connectivity (FC) analysis to explore the difference of interregional activity. Pearson's correlation analysis was conducted between rs-fMRI data and clinical symptoms in patients with PDM.

**Results:**

Compared with HCs, patients with PDM showed altered intraregional activity in a series of brain regions, including the hippocampus, the temporal pole superior temporal gyrus, the nucleus accumbens, the pregenual anterior cingulate cortex, the cerebellum_8, the middle temporal gyrus, the inferior temporal gyrus, the rolandic operculum, the postcentral gyrus and the middle frontal gyrus (MFG), and altered interregional FC mainly between regions of the mesocorticolimbic pathway and regions associated with sensation and movement. The anxiety symptoms are correlated with the intraregional activity of the right temporal pole superior temporal gyrus and FC between MFG and superior frontal gyrus.

**Conclusion:**

Our study showed a more comprehensive method to explore changes in brain activity in PDM. We found that the mesocorticolimbic pathway might play a key role in the chronic transformation of pain in PDM. We, therefore, speculate that the modulation of the mesocorticolimbic pathway may be a potential novel therapeutic mechanism for PDM.

## 1. Introduction

Primary dysmenorrhea (PDM) is defined as painful menstrual cramps without any pelvic pathology to account for them. The pain can radiate to the inner thighs and lower back and can be accompanied by symptoms such as nausea, vomiting, and diarrhea. A recent study showed that the prevalence of PDM among female college students in China can reach 41.7% (Hu et al., 2020), which demonstrates that PDM is the most prevalent gynecological problem among adolescent girls (Low et al., 2018).

Primary dysmenorrhea is formally coded and classified as chronic pain by the International Association for Study of Pain (IASP) and the World Health Organization (WHO) (Treede et al., 2015). Moreover, PDM often co-occurs with many other chronic pain conditions, including chronic headache, low back pain, irritable bowel syndrome, fibromyalgia, and painful bladder syndrome, with the highest prevalence rates after the age of 30 (Low et al., 2018), whereas the prevalence of PDM peaks before the age of 30 (Pan et al., 2014). In the past, the pathophysiological studies on PDM mainly focused on prostaglandins, antidiuretic hormones, and uterine blood flow, but none of them could fully explain the mechanism of PDM and the co-occurrence with other pain conditions later in life (Barcikowska et al., 2020; Tu and Hellman, 2021). In addition, PDM might induce some emotional problems, such as anxiety, as well as demonstrate increased pain sensitivity (Low et al., 2018) and allodynia (Giamberardino et al., 1997; Bajaj et al., 2002), whereas hyperalgesia (increased pain sensitivity) and allodynia are the two main characteristics of central sensitization (Sandkühler, 2009; Woolf, 2011). Notably, other pain conditions that co-occur with PDM, such as low back pain, irritable bowel syndrome, and fibromyalgia, often present central sensitization either (Arendt-Nielsen et al., 2018). In addition, individuals with PDM experience difficulties in the organization of dual-tasking and showed poorer postural stability performance (Keklicek et al., 2021), while postural control includes the integration of sensory-motor responses in the central nervous system (CNS) (Duarte and Freitas, 2010). To sum up, we have reasons to believe that neurobiological changes may play an important role in the occurrence and development of PDM.

Resting-state functional magnetic resonance imaging (rs-fMRI) records neural function using blood oxygen level dependency (BOLD) signals with important temporal characteristics. As a result, it is extensively used to investigate neurobiological changes in various disorders involving behavior, cognition, and pain. The changes in the brain structure and function of PDM have been demonstrated in several networks, including the default mode network (precuneus), reward network (nucleus accumbens), executive control network (dorsolateral prefrontal cortex), and attention network (anterior cingulate cortex), as well as some brain regions such as the thalamus and the periaqueductal gray matter (Jin et al., 2017; Liu et al., 2018; Low et al., 2018; Han et al., 2019; Zhang et al., 2019, 2020; Tu et al., 2021), most of which were based on regions of interest. For instance, Jin et al. (2017) studied regional homogeneity (ReHo) changes in dozens of regions of interest and found that abnormal ReHo value regions were mostly involved in the pain modulation network and DMN in the menstrual phase and sensorimotor processing and DMN in the non-menstrual phase. While in Wu's et al. (2016) research, no changes in ReHo values were found during menstruation in PDM. Hence, though there have been a number of studies on brain changes in PDM, they are insufficient and the results are inconsistent. A data-driven method that can more fully reflect brain activities might yield different findings.

Regional homogeneity (ReHo) measures the similarity of the time series of neighbor voxels according to Kendall's coefficient concordance (KCC), whereas the amplitude of low-frequency fluctuations (ALFF) measures the amplitude of time-series fluctuations at each voxel within a low-frequency range (Zuo et al., 2010), so the combination of the two can fully reflect intraregional spontaneous brain activity. Functional connectivity (FC) measures the correlation of temporal patterns of spontaneous neural activity between remote regions reflecting interregional functional connectivity (Fox and Raichle, 2007). The combination of the three methods fully reflects brain activity. Therefore, we used data-driven methods for the ReHo and ALFF analyses, and seed-based FC analyses were performed in the significant regions that were identified in the ReHo and ALFF analyses to reduce the uncertainty and bias of seed selection. We hypothesized that patients with PDM had altered spontaneous brain activities in a range of pain-related brain regions during menstruation, and some of these alterations may be associated with menstrual pain and related emotional symptoms.

## 2. Materials and methods

### 2.1. Subjects

Considering that PDM, as mentioned in the Introduction, mainly occurs before the age of 30 and has a high incidence among college students, our research subjects were recruited from Beijing universities. Thirty-three right-handed patients with PDM and 36 age-matched right-handed healthy controls (HCs) were recruited. All participants were fully informed of the project plan and signed informed consent. Patients with PDM should meet the diagnostic criteria issued by the Canadian Society of Obstetrics and Gynecology (Burnett and Lemyre, 2017). Inclusion criteria for the PDM group included no childbearing, regular menstrual cycles (28 ± 7 days), visual analog scale for pain (VAS-P) ≥40 mm, and without any treatment for the last three menstrual cycles. The HCs also had regular menstrual cycles and no reproductive history. The exclusion criteria for both patients with PDM and HCs included other pain-related diseases, history of mental disease, any magnetic resonance imaging (MRI) contraindications, and intracranial organic lesions.

### 2.2. Clinical measures

The clinical data were collected before the MRI scan, including the onset age and history of PDM and VAS (Steiner and Norman, 2016) 0 = no pain/anxiety sensation, 100 = the worst pain/anxiety sensation), which was used to evaluate the instant menstrual pain (VAS-P) and anxiety accompanied with pain (VAS-A).

### 2.3. MRI data acquisition and processing

All subjects underwent an MRI examination on the first–third day of their menstrual cycle (menstrual phase) using a Siemens Skyra 3-Tesla scanner with a head and neck combined 20-channel coil while keeping their eyes closed and awake. T2-weighted images were first performed to exclude intracranial lesions, then, high-resolution T1-weighted images and functional images (echo-planar imaging sequence, EPI) were collected in turn. The parameters of the MRI scan are shown in Table 1.

**Table 1:** Parameters of magnetic resonance scanning.

	**T2**	**T1**	**EPI**
TE (ms)	99	2.32	30.00
TR (ms)	4,000	2,300	3,000
FA (°)	150	8	90
MZ	384 × 384	256 × 256	94 × 94
FOV (mm^2^)	220 × 220	240 × 240	220 × 220
VZ (mm^3^)	0.6 × 0.6 × 5	0.9 × 0.9 × 0.9	2.3 × 2.3 × 3.0
Slices	20	192	40
Measurements	NA	NA	250

TE, echo time; TR, repetition time; FA, flip angle; MZ, matrix size; FOV, field of view; VZ, voxel size.

All 69 participants completed the questionnaires and MRI examination. Data preprocessing for ReHo, ALFF, and FC analyses were implemented on MATLAB platform (2021) by using Data Processing Assistant for Resting-State fMRI (DPARSF) v.4.4 (Yan et al., 2016) with statistical parametric mapping (SPM) 12 (http://www.fil.ion.ucl.ac.uk/spm, accessed on 3 September 2022). The common preprocessing includes standardized processing steps as follows: remove the first 10 time points; slice-timing and realignment were carried out for the remaining 240-time points with the exclusion criteria of 3 mm and 3 degrees in any direction (the middle slice was used as a reference), and all 69 data were eligible; normalize to Montreal Neurologic Institute (MNI) space using the DARTEL methods and resampled to 3 mm voxels; meanwhile, Friston 24 head motion parameters, cerebral spinal fluid signal, white matter signal, and global signal were removed as nuisance covariates; remove linear drift; filter with 0.01–0.08 Hz temporal bandpass. Preprocessing for ALFF and FC analysis also requires smoothing [a three-dimensional (3D) Gaussian kernel (full width at half maximum (FWHM) = 6 mm)], while preprocessing for ReHo analysis does not.

Regional homogeneity analysis was performed by calculating KCC as mentioned in the Introduction and smoothed after to allow the data to be normalized. The mean ALFF (mALFF) value was generated to standardize the data because the scale of the BOLD signal can affect the ALFF value. Functional connectivity analysis was performed using the method of seed-to-voxel FC analysis. The brain regions showing group differences in ReHo and ALFF analysis were used as the seed regions to further investigate interregional brain activity changes in patients with PDM. For each subject, Pearson's correlation analysis was performed between the mean time series of the seed and the time series of every voxel of the whole brain. Fisher's *r*-to-*z* transformation was followed to improve the normality of the correlation coefficients.

### 2.4. Statistical analysis

SPSS Statistics, version 22 software was used to analyze the demographic and clinical data, including age, menstruation days, menstrual cycle, history of PDM, onset age of PDM, VAS-P, and VAS-A. All the data were expressed as mean ± standard deviation (normal distribution), except the menstruation days described as minimum and maximum. A two-sample *t*-test was used for comparison between groups, and a level of *p* < 0.05 was considered statistically significant.

Two-tailed two-sample *t*-test [voxel threshold: uncorr. *p* < 0.001, cluster threshold: *p*-family-wise error (FWE) < 0.05] was performed to identify the ReHo, ALFF, and FC differences between the two groups, with the age, menstrual cycle, and menstrual day as covariates to be regressed.

Correlation analysis was applied using SPSS with a significance level of *p* < 0.05 to detect the underlying relationship between neuroimaging data showing group differences and clinical measures including the history of PDM, onset age of PDM, VAS-P, and VAS-A in patients with PDM by calculating Pearson's correlation coefficient.

## 3. Results

### 3.1. Demographics and clinical measures

The demographics and clinical measures of the two groups are summarized in Table 2. There were no significant differences between the groups in age, menstrual cycle, and menstruation days (*p* > 0.05).

**Table 2:** Demographic and clinical characteristics of subjects.

**Characteristics**	**PDM patients** **(*n* = 33)**	**Healthy controls (*n* = 36)**	***T*-test (*p*-value)**
Age (year)	24.24 ± 2.525	24.25 ± 2.170	0.989
Menstrual cycle (day)	30.09 ± 3.086	29.31 ± 2.109	0.218
Menstrual days (day)	4~7	4~7	0.117
Onset age of PDM (year)	16.36 ± 2.881	NA	NA
History of PDM (year)	7.88 ± 3.389	NA	NA
VAS-P	65.03 ± 16.337	NA	NA
VAS-A	55.30 ± 21.504	NA	NA

Values are mean ± standard deviation (SD); the menstrual days are described as minimum and maximum. PDM, primary dysmenorrhea; VAS-P, visual analog scale for pain; VAS-A, visual analog scale for anxiety.

### 3.2. Rs-fMRI data

**Figure 1:**
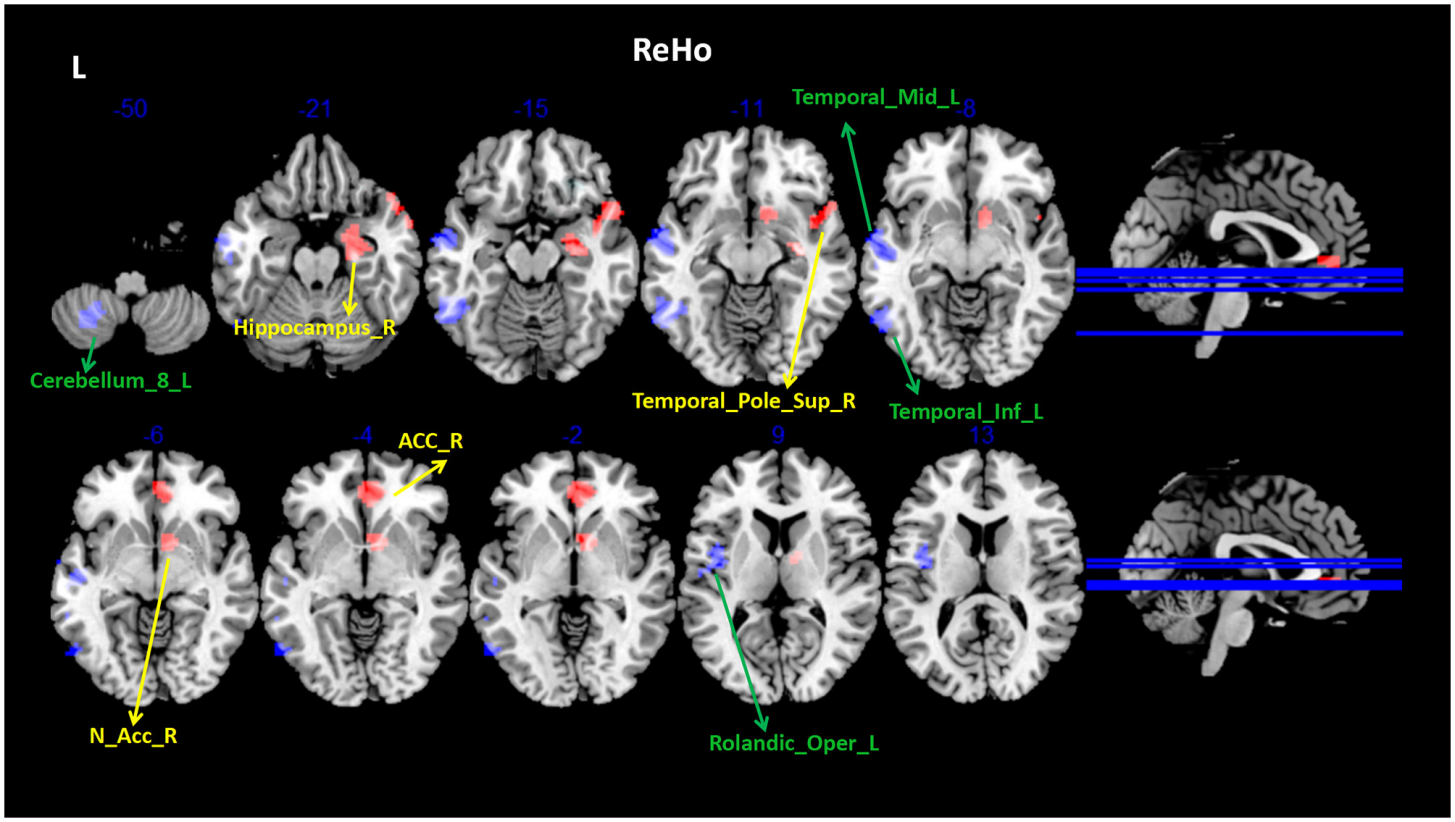
Group differences in ReHo between patients with PDM and HCs (voxel threshold: uncorr. *P* < 0.001, cluster threshold: *p*-FWE < 0.05). Patients with PDM had significantly higher ReHo values (yellow arrow) in the right hippocampus, temporal pole superior temporal gyrus, nucleus accumbens, and pregenual anterior cingulate cortex; and lower ReHo values (green arrow) in the left Cerebellum_8, middle temporal gyrus, inferior temporal gyrus, rolandic operculum. ReHo, regional homogeneity; PDM, patients with primary dysmenorrhea; HCs, healthy controls; FWE, family-wise error.

**Figure 2:**
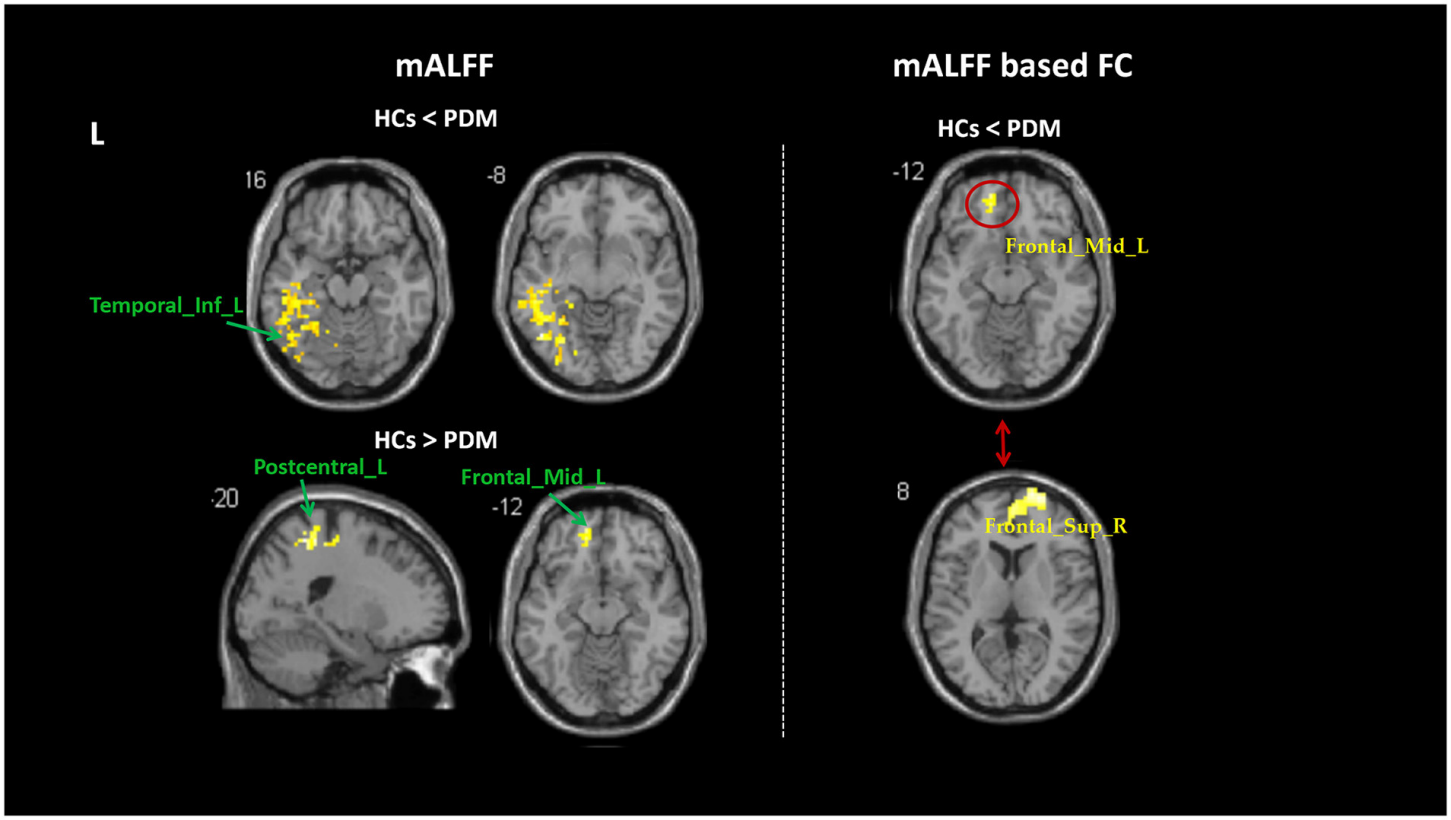
Group differences in mALFF and mALFF-based FC between patients with PDM and HC (voxel threshold: uncorr. *p* < 0.001, cluster threshold: *p*-FWE < 0.05). Patients with PDM had significantly increased mALFF values in the left inferior temporal gyrus and decreased mALFF values in the left postcentral gyrus and middle frontal gyrus. Patients with PDM showed enhanced FC between the left middle frontal gyrus and the right superior frontal gyrus. PDM, patients with primary dysmenorrhea; HCs, healthy controls; FWE, family-wise error; mALFF, mean amplitude of low-frequency fluctuations.

**Figure 3:**
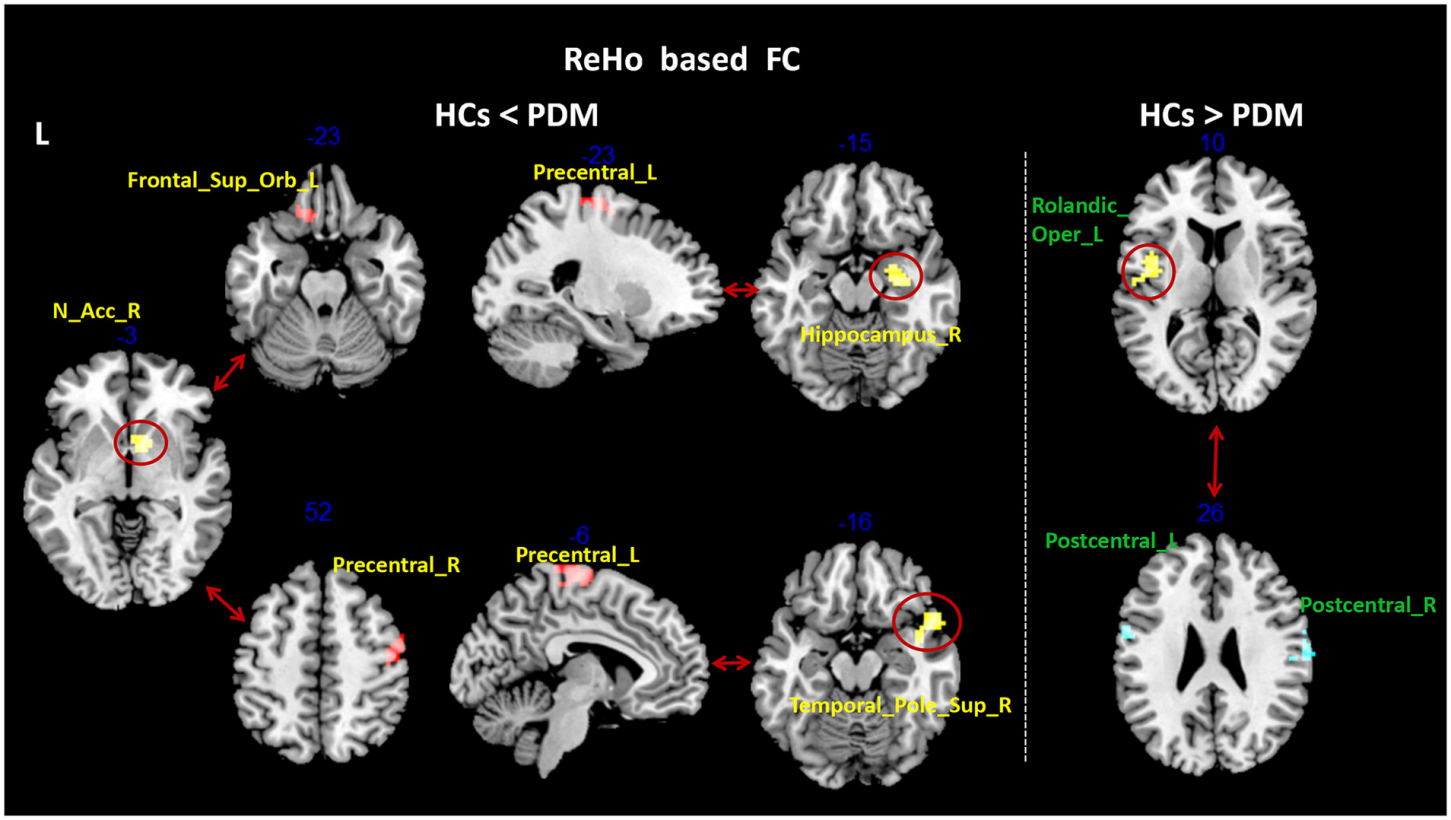
Group differences in ReHo-based FC between patients with PDM and HCs (voxel threshold: uncorr. *p* < 0.001, cluster threshold: *p*-FWE < 0.05). Patients with PDM showed enhanced FC between the right hippocampus and the left precentral gyrus, the right nucleus accumbens and the left supraorbital frontal gyrus and the right precentral gyrus, the right temporal pole superior temporal gyrus and the left precentral gyrus; and weakened FC between the left rolandic operculum and bilateral postcentral gyrus. PDM, patients with primary dysmenorrhea; HCs, healthy controls; FWE, family-wise error; ReHo, regional homogeneity; FC, functional connectivity.

Compared with the HCs, patients with PDM had significantly higher ReHo values in the right hippocampus (Hip), temporal pole superior temporal gyrus, nucleus accumbens (NAc), and pregenual anterior cingulate cortex (ACC), and lower ReHo values in the left Cerebellum_8, middle temporal gyrus (MTG), inferior temporal gyrus (ITG), and rolandic operculum (Figure 1 and Table 3). Patients with PDM had significantly increased mALFF values in the left inferior temporal gyrus and decreased mALFF values in the left postcentral gyrus (PoCG) and the middle frontal gyrus (MFG) (Figure 2 and Table 3). Compared with the HCs, patients with PDM showed enhanced FC between the right hippocampus and -the left precentral gyrus (PrCG), the right nucleus accumbens and -the left supraorbital frontal gyrus and -the right precentral gyrus, the right temporal pole superior temporal gyrus and -the left precentral gyrus, the left middle frontal gyrus and -the right superior frontal gyrus (SFG), and weakened functional connectivity between the left rolandic operculum and -the bilateral postcentral gyrus (Figures 2, 3 and Table 4). The brain regions were described according to the anatomical automatic labeling (AAL) templates.

**Table 3:** Brain regions showing ReHo differences between patients with PDM and HCs (voxel threshold: uncorr. *p* < 0.001, cluster threshold: *p*-FWE < 0.05).

	**AAL brain regions**	**MNI coordinates**			**Size (voxel)**	**Maximum *t*-value**
		***x***	***y***	***z***		
**ReHo**	**HCs** > **PDM**					
	Cerebellum_8_L	−30	−57	−51	66	5.6497
	Temporal_Mid_L	−54	−6	−15	178	4.9547
	Temporal_Inf_L	−54	−57	−12	138	4.7607
	Rolandic_Oper_L	−42	−3	9	68	5.3535
	**HCs** **<** **PDM**					
	Hippocampus_R	24	−6	−21	130	5.3678
	Temporal_Pole_Sup_R	57	18	−18	82	4.7557
	N_Acc_R	12	9	−9	131	4.5073
	ACC_pre_R	6	45	0	90	5.6433
**mALFF**	**HCs** > **PDM**					
	Frontal_Mid_L	−12	51	−12	46	4.5023
	Postcentral_L	−21	−48	57	156	5.246
	**HCs** **<** **PDM**					
	Temporal_Inf_L	−45	−63	−9	881	5.8007

PDM, primary dysmenorrhea; HC, healthy controls; FWE, family-wise error; ReHo, regional homogeneity; mALFF, mean amplitude of low-frequency fluctuations; MNI, Montreal Neurological Institute; AAL, anatomical automatic labeling; R, right; L, left.

**Table 4:** Regions showing functional connectivity differences between patients with PDM and HCs (voxel threshold: uncorr. *p* < 0.001, cluster threshold: *p*-FWE < 0.05).

**Seed regions**	**AAL brain regions**	**MNI coordinates**			**Size (voxel)**	**Maximum *t*-value**

		***x***	***y***	***z***		
Rolandic_Oper_L (HCs > PDM)	Postcentral_L	−63	−6	12	65	4.4028
	Postcentral_R	54	6	45	114	4.669
Hippocampus_R (HCs **<** PDM)	Precentral_L	−21	−18	66	70	4.5955
N_Acc_R (HCs **<** PDM)	Frontal_Sup_Orb_L	−18	30	−21	64	4.8912
	Precentral_R	51	−9	51	77	4.5543
Temporal_Pole_Sup_R (HCs **<** PDM)	Precentral_L	−9	−21	75	238	4.6257
Frontal_Mid_L (HCs **<** PDM)	Frontal_Sup_R	6	60	6	209	4.6015

PDM, patients with primary dysmenorrhea; HCs, healthy controls; FWE, family-wise error.

### 3.3. Correlation between MRI data and clinical measures

**Figure 4:**
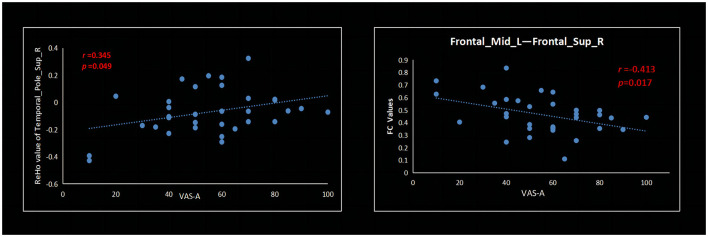
Analysis through Pearson's correlation: VAS-A is positively correlated with increased ReHo values of the right temporal pole superior temporal gyrus and negatively correlated with FC values between the left middle frontal gyrus and the right superior frontal gyrus. VAS-A, visual analog scale for anxiety.

In patients with PDM, the ReHo values of the right temporal pole superior temporal gyrus positively correlated with VAS-A (*r* = 0.345, *p* = 0.049) (Figure 4). The FC between the left middle frontal gyrus (MFG) and the right superior frontal gyrus (SFG) negatively correlated with VAS-A (*r* = −0.413, *p* = 0.017) (Figure 4).

## 4. Discussion

To our knowledge, the present study is the first to explore the alterations of brain spontaneous activity, simultaneously applying the method of ALFF, ReHo, and FC. These three methods reflect brain activity from different perspectives, so the combined application will give us more comprehensive information. The data-driven approach that we take can avoid bias caused by assumptions based on the area of interest and differences caused by different atlas or coordinate selections of the area of interest. In the present study, we identified altered intraregional brain spontaneous activity and FC in the mesocorticolimbic pathway, the pain sensorimotor brain regions, and the temporal lobe, among which the right temporal pole superior temporal gyrus and PFC may be involved in the emotional dimension of pain.

### 4.1. Abnormalities in brain regions associated with sensation and movement

Harris (1999) proposed the sensorimotor theory of pain, in which sensorimotor incongruence in pathological pain should relate to altered cortical processing and pain. Across several pathological pain conditions, motor and sensory deficits can be associated with alterations in areas related to sensory processing, motor control, and sensory-motor integration including the primary somatosensory (S1) and motor (M1) cortices, the cerebellum, and the posterior parietal cortex (Kuner and Flor, 2016; Chang et al., 2018; Eken et al., 2018; Goossens et al., 2018; Solstrand Dahlberg et al., 2018; Gentile et al., 2020), and affect pain-related emotions, motor imagery, etc. (Vittersø et al., 2022).

In the present study, we observed increased FC between PrCG and -the hippocampus,—NAc and—temporal pole superior temporal gyrus; decreased FC between bilateral PoCG and the left rolandic operculum; decreased mALFF in the left PoCG; and decreased ReHo in the left cerebellum and the left rolandic operculum. The PrCG belongs to M1, and the cerebellum is known to be related to motion (Baumann et al., 2015), so the two regions may all be about the motor changes in patients with PDM, whose structural and functional changes were found in patients with PDM (Han et al., 2019; Zhang et al., 2019; Wu et al., 2021b). People with chronic pain have pain that usually negatively affects their ability to move (Breivik et al., 2006). It has also been observed in our study that patients with PDM often adopt a crouching posture when in pain. Moreover, PrCG plays a role in pain that goes beyond motion, which is consistent with the sensorimotor theory and supported not only by the enhanced FC between PrCG and brain regions associated with memory, reward, and emotion found in our study, which represents functional integration with these areas, but also other studies. For instance, Wei et al. demonstrated adaptive hyperconnectivity with the sensorimotor cortex during painful menstruation during menstruation in patients with PDM (Wei et al., 2016). Rocco's research indicated that individual patterns of corticomotor reorganization during acute pain appear to be related to symptom severity, with early corticomotor depression possibly reflecting a protective response (Cavaleri et al., 2021). Research on the immediate analgesic effect of acupuncture in patients with PDM demonstrated that FC between ACC and PrCG was related to the short-term analgesic effect of acupuncture treatment. In another study, different strategies for regulating positive emotions (involving attentional deployment, cognitive change, and response regulation) were found to all co-activate brain regions such as the PrCG and ACC.

Voxels of the left rolandic operculum cluster in the present study are mainly located in the posterior part of the insula, which is one of the main spinothalamic cortical targets and one of the first regions of the brain recorded to respond to harmful stimuli (Dum et al., 2009). In Garcia-Larrea's third-order theory of pain experience, the posterior part of the insula belongs to the first-order processing (a nociceptive cortical matrix), appears as a necessary entry to generate physiological pain experiences, and could keep activation during sleep, coma, and vegetative state (Garcia-Larrea and Peyron, 2013). PoCG includes the primary somatosensory (S1) and encodes the sensory identification of pain, such as intensity and location (Peyron et al., 2000). Both of insula and S1 are involved in the encoding of pain and are highly regulated by cognitive factors including attention, emotion, and previous experience that could modify the sensory processing of pain (Dunckley et al., 2007; Bushnell et al., 2013), for example, distractions tend to reduce pain by inhibiting activity in afferent sensory and orienting areas of the spinothalamic system (like the insula) (Bantick et al., 2002). The insular and PoCG are consistent in functional imaging studies in PDM and even the field of pain. For instance, cerebral blood flow in the right rolandic operculum and FC between S1 and anterior cingulate cortex (ACC) increased (Zhang et al., 2020) during menstruation in patients with PDM. In our study, the decrease in mALFF in PoCG, ReHo in the insula, and FC between the two areas may reflect sensory aspects of pain.

In addition, as mentioned in the Introduction section, PDM often co-occurs with many other chronic pain conditions, including somatic and visceral chronic pain, and patients with PDM often demonstrate increased pain sensitivity, indicating that sensorimotor incongruence is not limited to the soma but also the viscera. Above all, cortical reorganization was the proposed reason for sensorimotor incongruence in the painful body, specifically in the areas of S1 and M1 (Tanasescu et al., 2016; Chang et al., 2018; Goossens et al., 2018). Though the evidence cannot decide whether sensorimotor incongruence could arise first and lead to pain or on the contrary, sensorimotor incongruence is more relevant for understanding why pain persists rather than how it arises (Vittersø et al., 2022). The discovery of sensorimotor brain regions may help us understand the poorer postural stability performance in patients with PDM.

### 4.2. Mesocorticolimbic pathway and transition to chronic pain

In addition to sensorimotor abnormalities, the comorbidities with other chronic pain disorders and manifestations of central sensitization are more notable in patients with PDM. This suggests that long-term menstrual pain may cause a reorganization of brain function, leading to chronic pain. Pain perception, memory, learning, and emotion may be involved in this chronic process, while nociception, memory, and emotional learning occur simultaneously and relies on the mesocorticolimbic pathway (Baliki and Apkarian, 2015). A significant portion of the brain regions that we found altered activity in our study belongs to this pathway. The hippocampus and NAc belong to the mesolimbic pathway, and PFC and ACC belong to the mesocortical pathway. They constitute the mesocorticolimbic pathway system, one of the main dopamine pathways in the brain, which is responsible for motive, encoding value, reward, aversion, and learning process (Barroso et al., 2021). This pathway has recently been found to be associated with the occurrence, amplification, and persistence of chronic pain and its emotion-affective dimensions, and the increase of FC between PFC and NAc may be a key gated process controlling the transition to chronic pain, as the amount of information shared between PFC and NAc is significantly higher in patients who subsequently transition to chronic pain (Vachon-Presseau et al., 2016). The roles of these brain regions are described separately later.

Simultaneously changed activity in ACC and pain sensory conduction areas (insular cortex and SI) or FC between them has been demonstrated in various chronic pain conditions. For instance, the FC between the insula and ACC in patients with fibromyalgia under acute pain stimulation was increased (Ichesco et al., 2016), as well as the increase of FC between ACC and S1 in the menstrual period of PDM mentioned earlier (Zhang et al., 2020). In the nociceptive pathway theory proposed by Bliss, nociceptive information from somatic and visceral organs is conveyed indirectly to the ACC through at least three major projection systems including the thalamus, the amygdala, and other pain-related areas of the cortex (such as S1 and the insular cortex), and then the information is projected onto the prefrontal cortex (PFC) and other subcortical areas (Bliss et al., 2016). Perhaps that is why ACC was divided into the second-order perception matrix of pain in Garcia-Larrea's third-order theory, participating in top-down projection influencing the pain sensory areas, and modifying perception by changing the sensations received at the source, such as the posterior insula (Garcia-Larrea and Peyron, 2013). As for the specific role, ACC is a key brain region of the salience network and is mainly involved in attention and emotional processing (Menon and Uddin, 2010). Oxytocin in ACC can reduce neuropathic pain and anxiety by inhibiting long-term presynaptic enhancement (Li et al., 2021). ACC may be involved in pain processing by activating attention and regulating pain-related emotional distress. In addition, studies have shown that providing cognitive strategies that allow subjects to manipulate activity in ACC can reduce the intensity of experimental pain and reduce the level of persistent pain in patients with chronic pain (deCharms et al., 2005). On the one hand, it shows the key role of ACC in pain regulation. On the other hand, fMRI has the potential to be used as a tool to help patients activate pain regulation systems and better control pain.

It is worth mentioning that in studies in the field of pain, insula, S1, and ACC seem to be the most relevant brain regions for acute pain, whether in animal models or human studies (Da Silva and Seminowicz, 2019). The functional changes in the corresponding regions found in this study may be partly caused by the presence of menstrual pain.

We found decreased mALFF in the left MFG, increased FC between NAc and supraorbital frontal gyrus, the left MFG, and the right SFG. MFG, SFG, and supraorbital frontal gyrus belong to the prefrontal cortex (PFC). PFC is the core region of the third-order network that produces impressive changes in the experience of pain by the modification of immediate percepts driven by the first and second-order pain matrix (Garcia-Larrea and Peyron, 2013). Not only is it closely linked to subcortical regions (especially periaqueductal gray matter, PAG) that are critical for the downward control of pain, supporting modifications in the subjective value of pain stimuli, but also it helps to create a cycle that changes the activity of the ascending nociceptive system; and through this bias, influence pain input (Garcia-Larrea and Peyron, 2013; Leknes et al., 2013). The increased FC between NAc and supraorbital frontal gyrus in our study may be the enhancement of top-down inhibition of pain. In a study on PDM acupuncture, it was also found that FC changes in the FC between MFG and PoCG were associated with acupuncture treatment (Tu et al., 2021). Increased FC between the left MFG and right SFG and negative correlation with VAS-A in our study seem to indicate that PFC is associated with pain anxiety in patients with PDM. Such results are also confirmed in the rat model of chronic neuropathic pain, as the reduction in the gray matter volume of the PFC occurs several months later and is associated with the occurrence of anxiety-like behaviors (Seminowicz et al., 2009). PFC reevaluates based on emotion and memory and participates in higher-order processing of pain, and structural and functional abnormalities are found in most pain studies (Garcia-Larrea and Peyron, 2013).

Patients with PDM had increased ReHo value in the hippocampus in the present study, which is consistent with previous studies (Jin et al., 2017). The hippocampus is an important part of memory formation and learning, and it is possible that the occurrence of chronic pain depends on memory consolidation in the hippocampus. Changes in hippocampal structure have been found in chronic pain (Coppola et al., 2017), which are related to pain memory (Berger et al., 2018). Patients with chronic pain also have obvious defects in hippocampal-related behaviors (Dick and Rashiq, 2007). Drugs injected into the hippocampus can reverse hypersensitivity to pain in rodents (Wei et al., 2021). We can understand that a complex sensory experience (like pain) involving cognitive, emotional, and interoceptive control relies on an individual's pain history and memory. The discovery of the hippocampus suggests the involvement of memory in the menstrual pain of patients with PDM. NAc is the core of the reward network. Pain relief can be interpreted as a reward. The reward circuit is closely related to the emotional circuit and overlaps significantly, and the gradual involvement of these two circuits suggests a shift away from acute pain and toward chronic pain (Hashmi et al., 2013).

Previous studies on PDM have also reported activity and structural changes in the mesocorticolimbic pathway. For instance, changes in network stability and variability of PFC, ACC, and Hip were found during pain-free periods, and these traits are associated with positive emotions and a sense of control (Wu et al., 2021a). In the pain-free stage, the ALFF of PFC and ACC increased in patients with PDM, and the ALFF of PFC was correlated with the course of the disease. All of these support our finding that the mesocorticolimbic pathway plays a crucial role in the chronic transformation of pain in patients with PDM.

### 4.3. Cerebellum and temporal area outside the medial temporal lobe cannot be ignored

Although we found substantial evidence that changes in the mesocorticolimbic pathway and sensorimotor region correspond to clinical manifestations of PDM, the findings in the cerebellar and temporal lobes seem less easily interpreted. Functional changes were found in the temporal lobe region except for the medial temporal lobe (including the hippocampus) in the present study, especially both ALFF and ReHo of the left MTG changed, and ReHo value of the temporal superior region was related to VAS-A, which seemed to indicate that this region is a very important one related to PDM and was involved in the processing of pain-related emotion (anxiety). However, considering the *p*-value of 0.049, the correlation needs to be further verified. The truth is that structural and functional changes in this region have been increasingly found in various pain-related diseases (Amin et al., 2021; Bunk et al., 2021). The thickness of temporal pole cortices during the attack compared to the inter-ictal phase is reduced in patients with migraine (Amin et al., 2021). Phase-amplitude coupling changes in the temporal lobe, especially the superior temporal region, were observed to be related to emotional prosody processing in patients with PDM (Chan et al., 2021). In addition, the increase of the ReHo value in the middle temporal gyrus was observed in both menstrual and non-menstrual periods (Jin et al., 2017), and the changes in FC between the temporal cortex and the thalamus were found in the ovulation period of PDM (Han et al., 2019). Particularly, gray matter features within the superior temporal gyrus during the periovulatory phase exhibited good performance in discriminating patients with PDM from HCs (Chen et al., 2019). Previous studies have shown that the temporal lobe plays an important role in memory or emotional processing (Marshall et al., 2019), but few studies have focused on its impact on harmful stimulus processing. Evidence of a link between this area and pain (especially pain-related emotion) remains elusive, and more evidence is needed.

The cerebellum may be an aggregator of multiple effector systems involved in various aspects of emotional processing, learning, memory, pain regulation, and sensorimotor processing (Stoodley and Schmahmann, 2010; Claassen et al., 2017; Michelle Welman et al., 2018; Brissenden and Somers, 2019; Slutsky-Ganesh et al., 2022). Both acute and chronic pain studies have found changes in the posterior cerebellum (Li et al., 2018; Zhang et al., 2019; Wu et al., 2021b; Slutsky-Ganesh et al., 2022). In addition, both during menstruation and ovulation, changes in intraregional activity and FC of the cerebellar posterior lobe were found in patients with PDM (Zhang et al., 2019; Wu et al., 2021b). Posterior cerebellar lesions may be associated with attention deficit, affective alterations, and reduced emotional expressivity by depriving cerebrum–cerebellar–cognitive limbic loops of cerebellar input (Stoodley and Schmahmann, 2010; Striemer et al., 2015; Sokolov et al., 2017). These studies support our findings of reduced ReHo in the posterior cerebellum. However, more research is needed to discover how the cerebellum is exactly involved in the pain process.

The highlight of our study was the alteration of activity in the brain regions most involved in the mesocorticolimbic pathway in patients with PDM, which was a new finding. Considering that our patients have had long-term menstrual pain (7.88 ± 3.389 years), we speculate that this may be related to pain chronicity. However, how to determine that the chronicity of pain has occurred still needs to be explored in future by longitudinal studies. Then, another important research question is how to elucidate the specificity of our findings for PDM since this pathway is also altered in chronic back pain as we mentioned, and studies that the modulating activity of brain regions in this pathway may help to address this question. Another question worth raising is that we chose ALFF, ReHo, and FC methods to explore the brain changes in PDM. Although the combination of the three methods seems to be more comprehensive than the single method, the significance of the specific brain activity reflected by these different indicators is still unclear and needs to be further clarified.

## 5. Conclusion

We found altered intraregional activity in a series of brain regions mainly involved in the mesocorticolimbic pathway, and altered interregional FC mainly between regions of the mesocorticolimbic pathway and regions associated with sensation and movement in patients with PDM with long-term menstrual pain. Given the cross-sectional nature of this study and the limited sample size, future longitudinal studies with a large sample size will help elucidate the specific mechanism of the mesocorticolimbic pathway changes in the occurrence of chronic pain in PDM. In conclusion, our study suggests that the modulation of the mesocorticolimbic pathway may be a potential novel therapeutic mechanism for PDM.

## Data availability statement

The raw data supporting the conclusions of this article will be made available by the authors, without undue reservation.

## Ethics statement

The studies involving human participants were reviewed and approved by Medical and Experimental Animal Ethics Committee of Beijing University of Chinese Medicine (Ethics Number: 2015BZHYLL0112) and Ethics Committee of Beijing University of Chinese Medicine (Ethics Number: 2021BZYLL03013). The patients/participants provided their written informed consent to participate in this study. Written informed consent was obtained from the individual(s) for the publication of any potentially identifiable images or data included in this article.

## Author contributions

Conceptualization and methodology: NL, YL, YHu, WL, and YZ. Software, writing, reviewing, and editing: WL and YZ. Validation: YHu and YZ. Formal analysis: TC and HW. Investigation, resources, and writing the original draft preparation: NL and YL. Data curation: YHo and JH. Visualization: NL, YL, YHo, and JH. Supervision: TC. Project administration: WL. Funding acquisition: YHu. All authors have read and agreed to the published version of the manuscript.
